# CIC-Related Neurodevelopmental Disorder: A Review of the Literature and an Expansion of Genotype and Phenotype

**DOI:** 10.3390/genes15111425

**Published:** 2024-10-31

**Authors:** Ivan Ruiz, Kimberly Wiltrout, Coral Stredny, Sonal Mahida

**Affiliations:** 1 Department of Neurology, Boston Children’s Hospital, Boston, MA 02115, USA; ivan.ruiz@childrens.harvard.edu (I.R.); kimberly.wiltrout@childrens.harvard.edu (K.W.);; 2 Harvard Medical School, Harvard University, Boston, MA 02115, USA

**Keywords:** CIC, epilepsy, neurodevelopment, developmental delay, intellectual disability, autism

## Abstract

Background: Genetic testing for neurodevelopmental disorders is now considered the standard of care for unexplained epilepsy as well as autism spectrum disorders, intellectual disability, and developmental delays with as many as 50% of individuals identified as having an underlying genetic etiology. Capicua (*CIC*) is a transcriptional repressor and is widely expressed among human brain tissue. Patients in the literature with pathogenic variants in *CIC* present with a broad spectrum of phenotypic abnormalities. Common features include epilepsy, developmental delay, intellectual disability, autism spectrum disorder, and MRI abnormalities amongst other neurodevelopmental symptoms. Variant type, age of onset, sex, and severity of manifestation also differ amongst probands. However, the full genotypic and phenotypic spectrum of *CIC*-related neurodevelopmental disorder has not been elucidated. Methods: Here we review patients reported in the literature with *CIC* variants and present two additional patients representing a novel genotype and phenotype. Results: Whole exome sequencing (WES) in this proband identified a novel paternally inherited likely pathogenic variant in *CIC* c.1526del p.(Pro509Hisfs*14). Both proband and father present with isolated epilepsy without other significant neurodevelopmental disorders. A review of the previous literature identified 20 individuals harboring *CIC* variants; the majority of these individuals present with a combination of neurodevelopmental features. Sixteen distinct variants were identified amongst these 20 patients. Conclusions: This family represents an expansion of the genotypic and phenotypic spectrum of *CIC*-related neurodevelopmental disorder. This information may lead to clinically actionable management changes for future patients identified with CIC variants considering standard anti-epileptic medication-weaning protocols.

## 1. Introduction

It is thought that up to two-thirds of otherwise unexplained epilepsies have an underlying monogenic cause [1]. Genetic testing for neurodevelopmental disorders (NDD), including unexplained epilepsy, is now considered to be the standard of care with recent guidelines suggesting whole exome and/or whole genome as the first tier test for many indications. This holds true even for those individuals with well-controlled, isolated epilepsy without other neurodevelopmental delays [2]. Historically, genetic testing was reserved for those individuals with severe developmental and epileptic encephalopathies (DEE). As epilepsy gene discovery has advanced, more individuals with self-limiting and isolated epilepsies are being offered genetic testing due to mounting evidence on the impact of genetic testing on treatment choices, prognosis, support resources, and access to clinical trials and research studies. Genetic testing is also part of the clinical workflow for unexplained autism spectrum disorder (ASD), intellectual disability (ID), developmental delay (DD), and other NDDs. Around 25–50% of cases of nonsyndromic ID are thought to have an underlying genetic etiology, with that figure being even higher for syndromic presentations [3]. Diagnoses of global developmental delay (GDD) and ID can be phenotypically and genotypically heterogeneous, but it is estimated that underlying genetic factors contribute to around 40% of developmental delays as well [4]. Thus, for patients with unexplained NDDs, genetic testing can be a useful tool to help identify an etiology and provide information on prognosis, treatment, management, and recurrence risks. As new gene–disease relationships are established for NDDs, the importance of genetic testing will continue to rise.

*CIC* (OMIM 612082) is a gene located on chromosome 19 (19q13.2) in humans. Expression data show that *CIC* is widely expressed throughout the brain, though most highly expressed in the cerebellum, hippocampus, and olfactory bulb [5]. The gene contains two isoforms—a short isoform (*CIC*-S) that is 1608 amino acids long, and a long isoform (CIC-L) that is 2514 amino acids long [6]. *CIC* is highly conserved across mammals. The gene was first discovered in Drosophila as a regulator of embryonic patterning with human and murine orthologs identified shortly after. *CIC* encodes the capicua protein, which is primarily involved in the repression of genes regulated by the RTK/Ras signaling pathway. It represses transcription through a complex known as the ATXN1-CIC complex. Disruption of the ATXN1-CIC complex in mice, either through *CIC* or *Atxn1-Atxn1L* knockout, results in behavioral deficits [7]. CIC also binds to the promoter regions of folate transport genes *FOLR1*, *PCFT*, and *SLC19A1*. Functional data show decreased FOLR1 expression in mutant CIC cells relative to controls, and this decreased FOLR1 ultimately reduces the cells’ ability to bind folic acid, which is essential for fetal neurodevelopment [8].

Variants in *CIC* have been reported in patients with a spectrum of neurodevelopmental presentations [6,7,8,9,10,11,12,13]. In OMIM, the gene–disease assertion is intellectual developmental disorder, autosomal dominant 45 [14]. However, not all patients with pathogenic *CIC* variants have ID or DD. *CIC* variants have been reported in 20 probands to date with a spectrum of features including neural tube defects, spinal bifida, epilepsy, and ASD amongst other neurodevelopmental phenotypes. Sudden unexpected death in epilepsy (SUDEP), which occurs in 1 out of every 1000 patients with epilepsy, has not been reported in this cohort [15]. Only 20 patients have been reported, however, so the incidence of SUDEP, the life expectancy, and the full phenotypic spectrum of *CIC*-related disorders is likely not fully understood. Here, we present a proband and her affected father, both of whom harbor a likely pathogenic variant in *CIC* and present with isolated epilepsy without other significant neurodevelopmental histories. These individuals represent an expansion of the phenotype for *CIC*-related neurodevelopmental disorder, which is critical for future variant interpretation and the identification of additional cases.

## 2. Materials and Methods

### 2.1. Literature Review

A comprehensive literature review was conducted for relevant *CIC*-related publications. Databases used included PubMed. Search keywords included CIC variant, CIC AND neurodevelopment, CIC AND epilepsy, CIC and inherited, and CIC AND gene function. Results were filtered by relevance based on titles and the screening of abstracts. A total of seven publications were used for the clinical literature review.

### 2.2. DNA Analysis

The DNA analysis section was adapted from GeneDx, Gaithersburg, MD, USA. The trio-based whole exome sequencing was completed by GeneDx laboratories, Gaithersburg, Maryland. The genomic DNA was extracted directly from the submitted specimen or, if applicable, from cultured fibroblasts. The DNA was enriched for the complete coding regions and splice site junctions for most genes of the human genome using a proprietary capture system developed by GeneDx for next-generation sequencing with CNV calling (NGS-CNV). The enriched targets were simultaneously sequenced with paired-end reads on an Illumina platform. Bi-directional sequence reads were assembled and aligned to reference sequences based on NCBI RefSeq transcripts and human genome build GRCh37/UCSC hg19. Using a custom-developed analysis tool (XomeAnalyzer), the data were filtered and analyzed to identify sequence variants and most deletions and duplications involving three or more coding exons [16]. Smaller deletions or duplications may not be reliably identified. Reported variants were confirmed, if necessary, by an appropriate orthogonal method in the proband and, if submitted, in selected relatives. Sequence variants are reported according to the Human Genome Variation Society (HGVS) guidelines. Copy number variants are reported based on the probe coordinates, the coordinates of the exons involved, or precise breakpoints when known. Reportable variants include pathogenic variants and likely pathogenic variants. Variants of uncertain significance, likely benign and benign variants, if present, are not routinely reported. The available evidence for variant classification may change over time and reported variant(s) may be reclassified according to the ACMG/AMP Standards and Guidelines [17], which may lead to issuing a revised report.

## 3. Results

### 3.1. Proband Medical History

The proband is a 20-year-old female followed in neurology clinic. The proband was born full-term via C-section, initiated due to large birth weight. She first presented to the neurology clinic at 2.5 months old with a concern for myoclonic movements in the extremities. These movements consisted of 1–2 min episodes that occurred intermittently, approximately a month apart. The workup at this time included a normal EEG with a diagnosis of benign sleep myoclonus. No additional testing was performed.

The patient returned to clinic at 18 years of age with absence seizures. Potentially relevant interim medical history includes constitutional delay of growth and puberty, anxiety, and early-onset hyperlipidemia.

**Figure 1:**
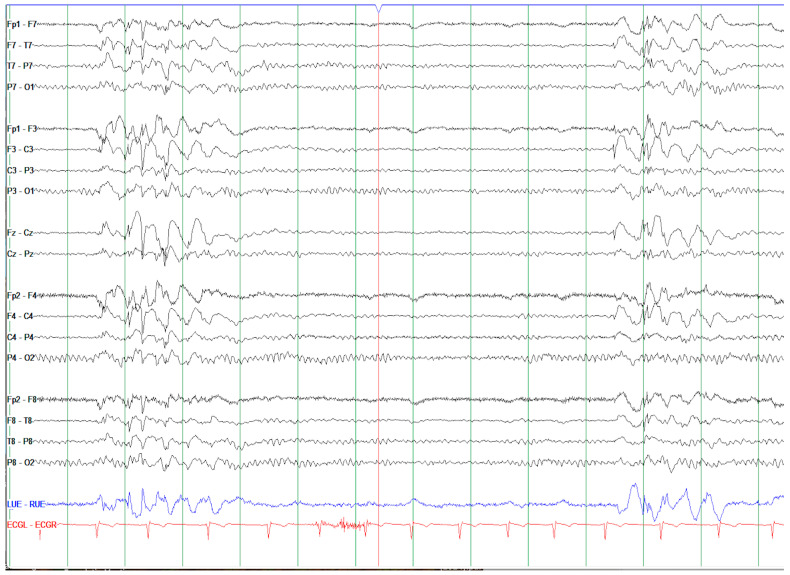
EEG of proband displayed in a longitudinal bipolar montage. Note bursts of generalized spike–wave discharges with bifrontal predominance (red box). During these 2–2.5 s bursts of generalized spike and polyspike and wave discharges, the patient displayed associated eye blinks or subtle pause/change in facial expression consistent with absence seizures. The EEG background was otherwise normal with an 11 Hz posterior dominant rhythm.

The workup at this stage included an EEG, which revealed bursts of generalized spike–wave discharges as well as at least nine 2–2.5 s bursts of generalized spike–wave discharges with associated eye blinks or subtle pause/change in facial expression suggestive of absence seizure (Figure 1). Keppra was initiated with good control. The official diagnosis was generalized epilepsy with absence seizures. Magnetic resonance imaging (MRI) of the brain was completed without contrast and revealed nonspecific scattered punctate FLAIR hyperintensities in the subcortical white matter, slightly elongated globes (likely associated with myopia), and a miniscule arachnoid cyst. These findings were interpreted as unremarkable and unrelated to the underlying etiology of epilepsy or migraine. In a recent exam, the patient was found to fit the diagnostic criteria for attention deficit hyperactivity disorder (ADHD), and reports possible migraines of temporal origin with photophobia/phonophobia and nausea, and a concern for anxiety and depression (no formal diagnoses). No learning disabilities or cognitive concerns are reported. The incidence rates of depression, anxiety disorders, ADHD, psychoses, and suicide are all significantly higher in patients with epilepsy compared to the general population [18]. While the mechanism behind these incident rates is unclear, in depression, for example, it is thought to be due in part to underlying neurological abnormalities and in part to the reality of living with epilepsy, where significant lifestyle changes are often made.

### 3.2. Family History (Figure 2)

The proband has one biological brother with a history of severe migraines with associated nausea and vomiting. The proband’s biological father has a diagnosis of generalized epilepsy initially presenting in the early teenage years. He is currently 54 years old and well-controlled on anti-seizure medication. Weaning of anti-seizure medications has been attempted, with significant breakthrough events. He also has a significant history of migraines as a child. The maternal family history is significant for migraines, but otherwise non-contributory.

Given the combination of the proband’s presentation and the family history of epilepsy with unknown etiology, trio whole exome sequencing (WES) was pursued.

### 3.3. CIC Variant

WES revealed a paternally inherited likely pathogenic truncating variant in *CIC* c.1526del p.(Pro509Hisfs*14). This variant is a predicted protein-truncating variant in a gene where loss of function (LOF) is a known mechanism of disease. The variant is not present in the gnomAD population database, allowing the variant to reach likely pathogenic classification. *CIC* has a pLI score of 1 (gnomAD 4.1), and a loss-of-function observed/expected upper bound fraction (LOEUF) of 0.25 [19]. The variant was not identified in the proband’s brother or mother.

### 3.4. POF1B Variant

WES also revealed a paternally inherited truncating variant of uncertain significance (VUS) in *POF1B* c.589del p.(Gln197Argfs*46). *POF1B* is located on the X-chromosome and has been shown to be involved in actin filament binding [20]. Currently, there is no evidence for association between variants in *POF1B* and neurodevelopmental disorders.

### 3.5. Phenotypic and Genotypic Spectrum

**Figure 3:**
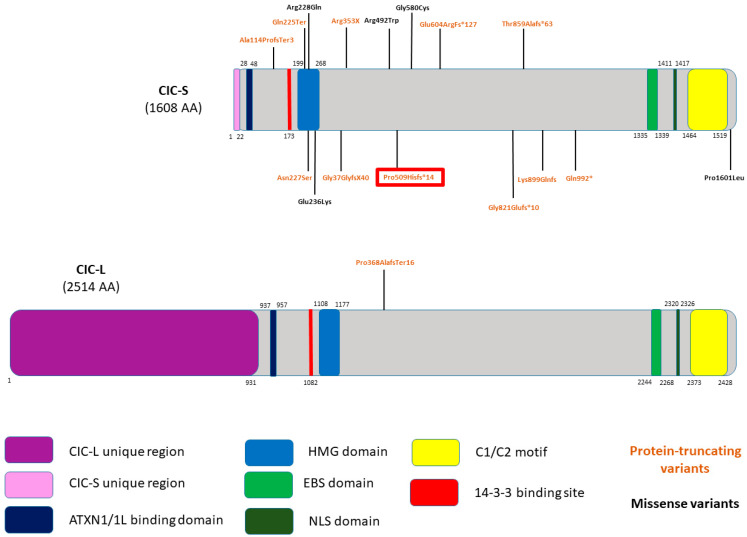
Previously published variants mapped across CIC protein domains. Novel identified variant in the present proband and her father are highlighted in red. Both CIC isoforms are represented with only one variant identified in the long isoform (CIC-L). Variant type can be distinguished by color with protein-truncating variants in orange and missense variants in black.

The literature review identified 16 distinct variants found across 20 individuals, none of which were present in gnomAD 4.1 (Table 1, Figure 3). Although one variant was identified in the CIC-L isoform, the other fifteen variants were found in the CIC-S isoform. Ten protein-truncating variants and six missense variants were identified. Furthermore, ten are substitution variants, three are deletion variants, and the last three variants include one insertion, one deletion, and one indel variant. Most variants are de novo (11/20), some are unknown inheritance (6/20), and few are inherited (3/20), one from a mosaic father. Three identified variants—*CIC* c.1100dup p.(pro386AlafsTer16), *CIC* c.673C>T p.(Gln225Ter), and *CIC* c.1057C>T p.(Arg353*)—have functional evidence to confirm pathogenicity [6,8].

Patients reported in the literature range in age, sex, age of symptom onset, severity of symptoms, and more (Table 2). We identified twelve male and eight female patients. The most common phenotypes across individuals with *CIC* variants are ID (15/20), GDD (8/20), and seizures/epilepsy (8/20). The publications did not detail any standardized cognitive testing for patients reported with ID or GDD. Other common findings for patients with CIC variants include ASD, MRI abnormalities, and cerebral folate deficiency. Most patients present with a combination of these neurodevelopmental features, though some present with only one distinguishing feature. No patients reported in the literature present with isolated epilepsy without other significant neurodevelopmental delays. Our family, father and daughter, represent a phenotypic expansion for CIC-related neurodevelopmental disorder with a presentation of isolated epilepsy without other significant neurodevelopmental delays.

## 4. Discussion

In this paper, we report on a proband and her father who were found to have a heterozygous likely pathogenic variant in *CIC* c.1526del p.(Pro509Hisfs*14). The proband’s clinical exam and MRI were unrevealing for significant underlying etiologies. The variant identified is predicted to be protein-truncating, further supporting evidence towards pathogenicity, though patients with missense and nonsense variants in *CIC* have been reported. This variant is especially significant given the constraint scores and metrics for *CIC*, a gene that is loss-of-function intolerant. The proband and her father also each harbor a heterozygous VUS in *POF1B*, seemingly unrelated to neurodevelopment at this time.

### 4.1. Phenotypic Variability of CIC Variants

The phenotypic spectrum for *CIC*-related neurodevelopmental disorder is characterized by variable expressivity. Cao et al.’s proband harbors a de novo truncating variant, c.1057C>T, p.Arg353*, presenting with the aforementioned cerebral folate deficiency [8]. Lu et al. reports a 16-year-old female with the same de novo truncating variant, presenting with global developmental delay, intellectual disability, autism, epilepsy, macrocephaly, and telangiectasia. An MRI for this patient also revealed punctate foci of T2 hyper intensity within the subcortical white matter [9].

Kishnani et al. reports a trio of affected family members with a wide spectrum of phenotypic presentations, despite having the same underlying variant [11]. WES identified a maternally inherited protein-truncating variant, c.2694dupC, (Lys899GlnfsX32). The proband is a 16-year-old female with a history of moderate language delays, ID, ASD, and intractable absence epilepsy with onset at 18 months of age. This proband’s brother, who also harbors the variant, has a history of absence epilepsy with onset at 7 years of age and his first convulsive seizure at 13 years old. Frequent seizures reportedly caused developmental stagnation and regression. The mother of both children presented with a single isolated seizure at age 13 years. She is well-controlled on valproic acid, and remains seizure-free. This family is a prime example of the variable expressivity seen within *CIC*-related neurodevelopmental disorders.

Of note, the three individuals in this family were also reported to have a VUS in *ANK3*, c.4052 T>C p.Val1351Ala. The variant is within a highly conserved region and is not present in gnomAD. In silico analysis predicts the variant may be damaging to the function of the protein. *ANK3* variants have been reported in patients with a spectrum of neurodevelopmental features including ASD, DD/ID, psychiatric disturbances, seizures, and more [21]. While the clinical significance of the variant is unclear, it may be contributing to the underlying etiology for the proband and family members’ phenotypes.

The mother reported in Kishnani et al. is the only other patient reported with epilepsy as the only clinically relevant symptom [11]. However, her genotype is confounded by the presence of the ANK3 variant. Our proband and her father are the first patients reported in the literature with isolated epilepsy and represent an expansion of the phenotype for *CIC*-related neurodevelopmental disorders.

### 4.2. Genotypic Variability of CIC Variants

Most variants in *CIC* reported in the literature thus far are protein-truncating (10/16); however, a clear genotype–phenotype correlation has not been elucidated. Functional studies seem to suggest that CIC haploinsufficiency is the underlying mechanism, which leads to altered function in cells [6]. As such, no individuals have been reported with compound heterozygous or homozygous *CIC* variants, indicating the potential lethality of biallelic pathogenic variants. Variants reported in the literature thus far span across various protein domains (Figure 3). The aforementioned phenotypic variability is likely in part due to the genotypic variability observed. Like many other neurodevelopmental disorders, variable expressivity is also likely a contributor to the phenotypic variability observed. Given this variability, special care should be taken to review variants inherited from seemingly unaffected parents.

### 4.3. Mechansim and Clinical Application

CIC functions primarily as a transcriptional repressor, repressing genes involved in cell proliferation, neurogenesis, and cell-cycle regulation. Consequently, *CIC* variants can lead to uncontrolled cell growth, which was observed in a patient with acute lymphocytic leukemia (ALL) [6]. While CIC haploinsufficiency results in the *CIC*-related neurodevelopmental disorder, the precise mechanism of pathogenicity remains unclear. CIC functions in the development of multiple systems including the central nervous system, immune system, and lungs. One plausible mechanism of pathogenicity is dysregulation of the RAS-MAPK signaling pathway, which is essential for proper neuronal functioning. NDDs caused by variants affecting this signaling pathway are known as RASopathies, including conditions such as Noonan syndrome or Neurofibromatosis type 1 [22]. The mechanism behind *CIC*-related neurodevelopmental disorder is likely similar to other RASopathies, though future research is needed to clarify this.

Both patients presented in this report have remained seizure-free on anti-seizure medication. For the proband’s father, even after years of seizure freedom, weaning off medications resulted in significant breakthrough events suggesting a high lifetime risk of epilepsy for patients with *CIC*-related neurodevelopmental disorder. This information may lead to clinically actionable management changes for future patients identified with CIC variants considering standard anti-epileptic medication-weaning protocols.

## 5. Conclusions

We report the first case of an inherited variant in two individuals with isolated epilepsy. As patients continue to be identified, our understanding of the nature of the genotypic and phenotypic diversity of *CIC* variants will continue to expand. This report may help clarify the spectrum reported and be of diagnostic utility for future patients. Future research efforts to clarify the mechanism of disease for *CIC* variants and implications for treatment and management are needed.

## Figures and Tables

**Figure 2:**
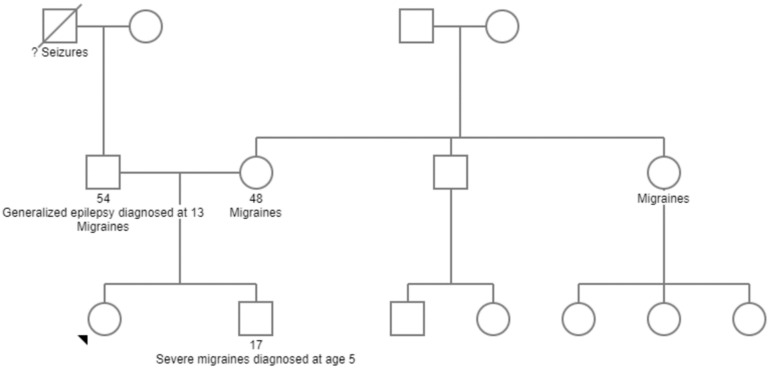
Three-generation pedigree showing proband (black arrow) and other affected family members with relevant medical histories.

**Table 1:** *CIC* variants identified in the literature review including variant type and predicted effect.

PMID	Variant	Inheritance	Type	Prediction
**Present case**	c.1526del p.(P509Hfs*14)	Paternally inherited	Deletion	Frameshift-inducing early stop
**35165976** [6]	c.1100dup p.(pro386AlafsTer16)	De novo	Duplication	Frameshift-inducing early stop
	c.673C>T p.(Gln225Ter)	De novo	Substitution	Nonsense-inducing early stop
	c.683G>A p.(Arg228Gln)	Unknown	Substitution	Missense variant
	c.3439_3440delGC p.Ala114ProfsTer3	De novo	Deletion	Frameshift-inducing early stop
**32820034** [8]	c.C1057T p.R353*,	De Novo	Substitution	Nonsense-inducing early stop
	c.1008_1009InsTG:p.G337GfsX40	De Novo	Insertion	Frameshift inducing early stop
	c.C4802T P1601L	Unknown	Substitution	Missense variant
	G1738T PG580C	Unknown	Substitution	Missense variant
**28288114** [9]	c.1057C>T, p.Arg353*	De novo	Substitution	Nonsense inducing early stop
	c.1801_1808dupAAGAGACC (p.Glu604ArgFs*127)	De novo (presumed germline)	Duplication	Frameshift-inducing early stop
	c.1801_1808dupAAGAGACC (p.Glu604ArgFs*127)	De novo (presumed germline)	Duplication	Frameshift-inducing early stop
	c.2571_2579delinsC p.Thr859Alafs*63	De novo	Indel	Frameshift-inducing early stop
	c.2974C>T Gln992*	Inherited from mosaic father	Substitution	Nonsense inducing early stop
**24307393** [10]	c.680A>G p.Asn227Ser	De novo	Substitution	Missense variant
**Kishnani et al. (No PMID)** [11]	c.2694dupC pK899QfsX32	Maternally inherited	Duplication	Frameshift-inducing early stop
	c.2694dupC pK899QfsX32	Maternally inherited	Duplication	Frameshift-inducing early stop
	c.2694dupC pK899QfsX32	Unknown	Duplication	Frameshift-inducing early stop
**21076407** [12]	c.1474C>T p.Arg492Trp	Unknown	Substitution	Missense
**30030131** [13]	c.706G>A p.Glu236Lys	De novo	Substitution	Missense
	c.2462delG p.Gly821Glufs*10	Unknown	Substitution	Frameshift-inducing early stop

**Table 2:** Phenotypic features of individuals identified in literature review.

PMID	Age	ASAB	GDD	Hypotonia	Speech delays	Developmental regression	ADD/ADHD	ID	ASD	Epilepsy	EEG	MRI
35165976 [6]	12	F	-	-	+	-	-	+	+	-		Within normal limits
	8	F	-	-		-	-	+		+	Generalized spike and wave and polyspikes	Within normal limits
	13	M	-	-	+	+	-	+	-	-		Abnormal, but due to chemotherapy for ALL
	12	M	+	+	+	-	+	-	+	-		
32820034 [8]	U	U	-	-	-	-	-	-	-	-		
	U	U	-	-	-	-	-	-	-	-		
	U	U	-	-	-	-	-	-	-	-		
	U	U	-	-	-	-	-	-	-	-		
28288114 [9]	16	F	+	-	-	-	-	+	+	+		Punctate foci of T2 hyperintensity within the subcortical white matter
	27	F	+	-	-	-	-	+	-	+		Within normal limits
	9	M	+	+	-	-	+	+	-	+		Within normal limits
	4	M	+	+	-	-	-	+	+	-		Puncate focus of T2 hyperintesnity in R frontal lobe white matter
	15	M	+	-	-	-	+	+	+	-		Several periventicular T2 hyperintensities in white matter
24307393 [10]	U	M	-	-		-	-	+				
Kishnani et al. (No PMID) [11]	16	F	-	-	+	-	-	+	+	+	Bursts of generalized spike and sharp waves, polyspikes	
	14	M	-	-	-	+	-	+	-	+	Frequent generalized bursts of spikes and polyspike wave discharges	Within normal limits
	U	F	-	-	-	-	-	-	-	+		
21076407 [12]	U	M	-	-	-	-	-	+	-	-		
30030131 [13]	U	F	+	-	-	-	-	+	-	-		
	U	F	+	-	-	-	-	+	-	-		

## Data Availability

No new data were created or analyzed in this study. Data sharing is not applicable to this article.
